# Supplementary material for: Osthole Prevents Heart Damage Induced by Diet-Induced Metabolic Syndrome: Role of Fructokinase (KHK)
Source: Antioxidants (Basel). 2023 Apr 28;12(5):1023. doi: 10.3390/antiox12051023 (PMC10215822; doi:10.3390/antiox12051023)
Supplement: Supplementary file 1 [file antioxidants-12-01023-s001.zip › antioxidants-2316451-supplementary.pdf]

Supplementary material

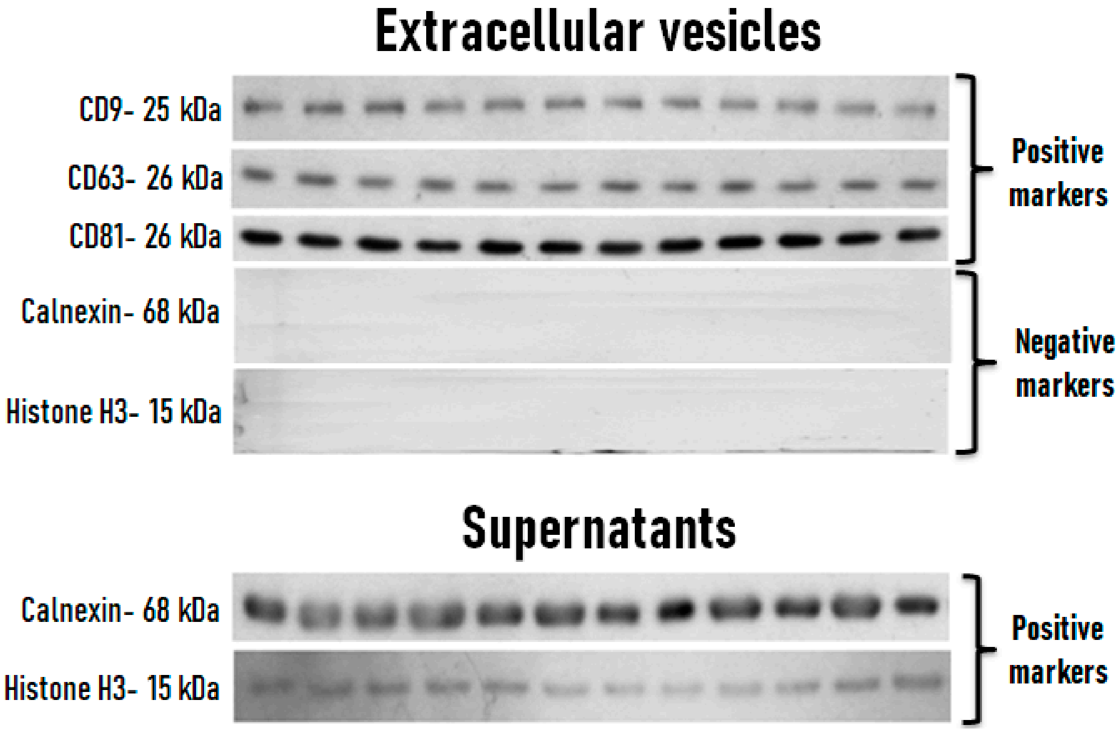

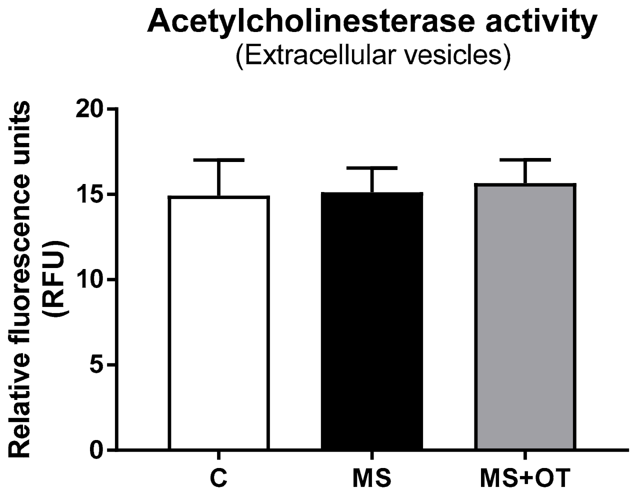

Supp Figure S1: Characterization of Extracellular vesicles by expression of positive and negative specific markers and AChE activity.

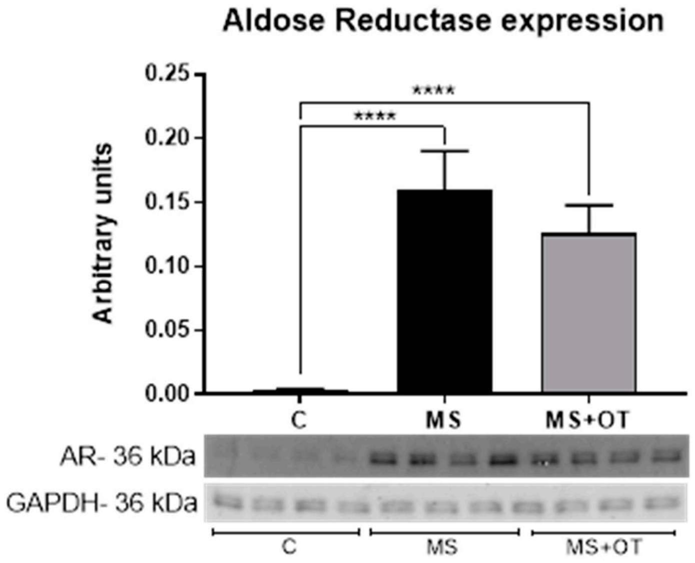

Suppl. Figure S2. Effect of Paigen type-diet and osthole administration in cardiac AR expression measured by WB after 30 days follow-up. Data are presented as the mean ± standard deviation and were analyzed by one-way ANOVA. Analysis post-hoc was performed using Tukey´s multiple comparison test. Statistical differences were
